# Evaluating In Silico the Potential Health and Environmental Benefits of Houseplant Volatile Organic Compounds for an Emerging ‘Indoor Forest Bathing’ Approach

**DOI:** 10.3390/ijerph19010273

**Published:** 2021-12-27

**Authors:** Valentina Roviello, Pasqualina Liana Scognamiglio, Ugo Caruso, Caterina Vicidomini, Giovanni N. Roviello

**Affiliations:** 1 Department of Chemical, Materials and Industrial Production Engineering (DICMaPI), University of Naples Federico II, Piazzale V. Tecchio 80, 80125 Naples, Italy; 2 Center for Advanced Biomaterial for Health Care (CABHC), Istituto Italiano di Tecnologia, 80125 Naples, Italy; pasqualina.scognamiglio@iit.it; 3 Department of Chemical Sciences, University of Naples Federico II, Via Cintia 21, 80126 Naples, Italy; ugo.caruso@unina.it; 4 Istituto di Biostrutture e Bioimmagini IBB-CNR, Via Tommaso De Amicis 95, 80145 Naples, Italy; caterina.vicidomini@ibb.cnr.it

**Keywords:** houseplant, biogenic volatile organic compounds, forest bathing, environmental, human health, in silico analysis, SARS-CoV-2, COVID-19, *Spathiphyllum wallisii*, *Aspidistra eliator*

## Abstract

The practice of spending time in green areas to gain the health benefits provided by trees is well known, especially in Asia, as ‘forest bathing’, and the consequent protective and experimentally detectable effects on the human body have been linked to the biogenic volatile organic compounds released by plants. Houseplants are common in houses over the globe and are particularly appreciated for aesthetic reasons as well for their ability to purify air from some environmental volatile pollutants indoors. However, to the best of our knowledge, no attempt has been made to describe the health benefits achievable from houseplants thanks to the biogenic volatile organic compounds released, especially during the day, from some of them. Therefore, we performed the present study, based on both a literature analysis and in silico studies, to investigate whether the volatile compounds and aerosol constituents emitted by some of the most common houseplants (such as peace lily plant, *Spathiphyllum wallisii*, and iron plant, *Aspidistra eliator*) could be exploited in ‘indoor forest bathing’ approaches, as proposed here for the first time not only in private houses but also public spaces, such as offices, hospitals, and schools. By using molecular docking (MD) and other in silico methodologies for estimating vapor pressures and chemico-physical/pharmacokinetic properties prediction, we found that β-costol is an organic compound, emitted in appreciable amounts by the houseplant *Spathiphyllum wallisii*, endowed with potential antiviral properties as emerged by our MD calculations in a SARS-CoV-2 M^pro^ (main protease) inhibition study, together with sesquirosefuran. Our studies suggest that the anti-COVID-19 potential of these houseplant-emitted compounds is comparable or even higher than known M^pro^ inhibitors, such as eugenol, and sustain the utility of houseplants as indoor biogenic volatile organic compound emitters for immunity boosting and health protection.

## 1. Introduction

Numerous are the benefits that spending time in a green area can provide from both a psychological and physical perspective [1], and this is the reason why the so-called ‘forest bathing’ [2,3] is gaining more and more popularity also outside its original area, Japan, together with the ‘green prescriptions’ [4], whose importance for patient care is being recognized in an increasing number of countries across the globe. The long-lasting effects of ‘forest bathing’ on body immunity and, more in general, on human health are associated with the inhalation of biogenic volatile organic compounds (VOC) and other plant-emitted aerosol constituents by individuals who frequent parks or forests, which trigger biological processes with health-protective effects [5,6,7,8]. Unfortunately, it is not always easy to find places suitable for the ‘forest bathing’ practice, especially in the context of megacities characterized by dense populations and scarcity of green spaces. On the other hand, indoor houseplants create comfortable environments inside houses and workplaces, where they improve concentration and productivity, reduce stress levels, and boost mood [9,10]. From an environmental perspective, houseplants can be useful for air purification due to their ability to remove some volatile pollutants [11]. In fact, those exposed to polluted indoor air may experience ‘new house syndrome’, ‘multiple chemical sensitivity’, and ‘sick building syndrome’, alongside several adverse physical symptoms such as frequent fatigue, headache, allergies, and asthma, to cite only a few [12]. Chemically, benzene, formaldehyde, and 2-ethyl-1-hexanol are common indoor pollutants emitted from a number of materials commonly present inside buildings that are harmful to our health [13]. Fortunately, houseplants such as peace lily plant (*Spathiphyllum wallisii*), but also ivy (*Hedera helix*), are able to lower the indoor concentration of toluene and benzene, providing an effective biofiltration [13]. Interestingly, these two common houseplants were proven to significantly remove also CO_2_ across a range of indoor light levels [14]. Notwithstanding the above reports, other recent studies, evaluating the anthropogenic VOC (AVOC) removal efficiencies of houseplants, have suggested that they do not lead to improved indoor air quality [15], an effect not in correlation with plant-emitted isoprene and O_3_ [16], not clarifying then which combined effect the plant VOC emissions, pollutants removal, and secondary byproducts, resulting from the interactions of the pollutants with the plant-emitted VOCs, could have on indoor air quality [17]. Nonetheless, the aim of the present work was not studying the ability of the specific houseplants herein mentioned to remove AVOC pollutants to clean the air indoors, but to show in silico that biogenic VOCs (BVOC) and aerosol constituents emitted by houseplants have potential therapeutic (and anti-COVID-19) activities.

In fact, houseplants release into the surrounding environments several biogenic volatile molecules, some of which play important roles in flavor and scent, and are endowed with precise biological roles, such as attracting pollinators, enhancing thermotolerance, and protecting against herbivories or plant pathogens [18,19]. Several plant-derived terpenoids, such as limonene, linalool, α-pinene, and β-thujone, contribute to the fragrance of houseplants, such as *Heliotropium arborescens* [20]. Although significant advances have been made in the identification of forest-emitted volatile organic compounds and the characterization of their health benefits, much less is known about the volatiles produced by indoor plants and, to the best of our knowledge, no attempt has been reported to use houseplants in ‘green therapies’ through simple inhalation of the emitted volatiles. The objective of this study was, thus, to review the literature on the main volatiles emitted by some common indoor plants and, by using an in silico approach, to evaluate their potential role on human health protection, especially as inhalable antiviral drugs to be used, for example, in the fight against SARS-CoV-2 that is causing the current COVID-19 pandemic [21].

## 2. Materials and Methods

### 2.1. Literature Analysis

The literature analysis was conducted on Medline/Pubmed and Google Scholar, using the terms ‘houseplants’, ‘biogenic volatile organic compounds’, and ‘volatile organic compounds’, excluding from the subsequent literature study those works reporting the use of house plant for removing anthropogenic volatile organic compounds, as we were interested specifically in the plant-released (biogenic) volatiles.

### 2.2. Molecular Docking

The three-dimensional structure of the protein target from SARS-CoV–2, i.e., the main protease M^pro^ (PDB ID: 6Y84), was obtained from Protein Data Bank [22]. The 2D structures for the ligands were retrieved from the PubChem database (https://pubchem.ncbi.nlm.nih.gov/, accessed on 17 November 2021). 1-Click Mcule (Mcule Inc., Palo Alto, CA, USA) [23,24,25], a web-based platform powered by the AutoDock Vina docking algorithm [26], was used for our docking experiments. The atomic coordinates of the binding site were those reported in the literature [27] (X: 9.204, Y: −4.557 and Z: 19.602), and the size of the binding site was 22 Angstrom. We selected the docking poses with the most negative docking scores (kcal/mol), corresponding to the highest binding affinities, for further structure visualizations and analyses. We validated the docking method applying it to other literature dockings targeting M^pro^, finding our binding energy scores in line with those previously reported for umbelliferone and eugenol [27]. Moreover, we obtained the protein–ligand interaction diagrams reported in this work by PLIP (Protein–Ligand Interaction Profiler, https://plip-tool.biotec.tu-dresden.de/, accessed on 17 November 2021) [28].

### 2.3. Prediction of Pharmacokinetic Properties and Vapor Pressures

The logarithms of the partition coefficients (cLogP), blood–brain barrier (BBB) permeability, pan-assay interference compounds (PAINS) score, and druggability properties shown in this work and in Supporting Information (Figures S1–S15) were predicted for the indoor plant-emitted organic compounds by SwissADME (http://www.swissadme.ch/index.php, accessed on 17 November 2021). Vapor pressures (at 25 °C) were calculated by UManSysProp (http://umansysprop.seaes.manchester.ac.uk/tool/vapour_pressure, accessed on 17 November 2021), using the ‘Nannoolal 2008’ vapor pressure method and the ‘Joback and Reid 1987’ boiling point method.

## 3. Results and Discussion

Our literature analysis showed that only few reports on houseplant volatile organic compounds are present in the literature, and that these rare examples are basically analytical works describing the composition in volatiles of emissions of specific houseplants. No example on use of houseplants in ‘forest-bathing’-like approaches was found.

### 3.1. Houseplant-Emitted Volatile Organic Compounds

**Figure 1:**
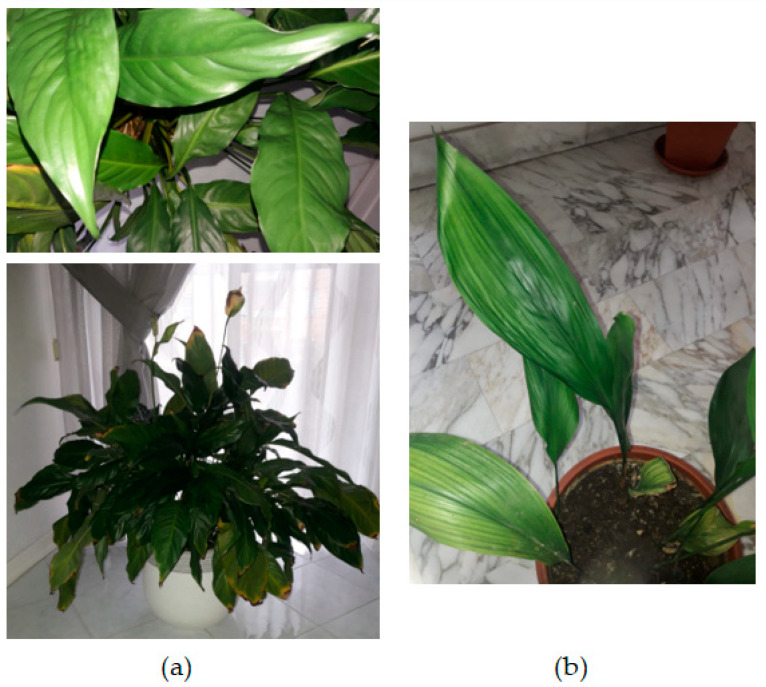
Closeup and plant view of (**a**) the peace lily plant (*Spathiphyllum wallisii*, left); and (**b**) green stems and leaves of the iron plant (*Aspidistra eliator*, right). Note that while the peace lily plant enjoys humidity conditions, the iron plant is named for its ability to survive a wide range of conditions, including drought, shade, and pests.

**Figure 2:**
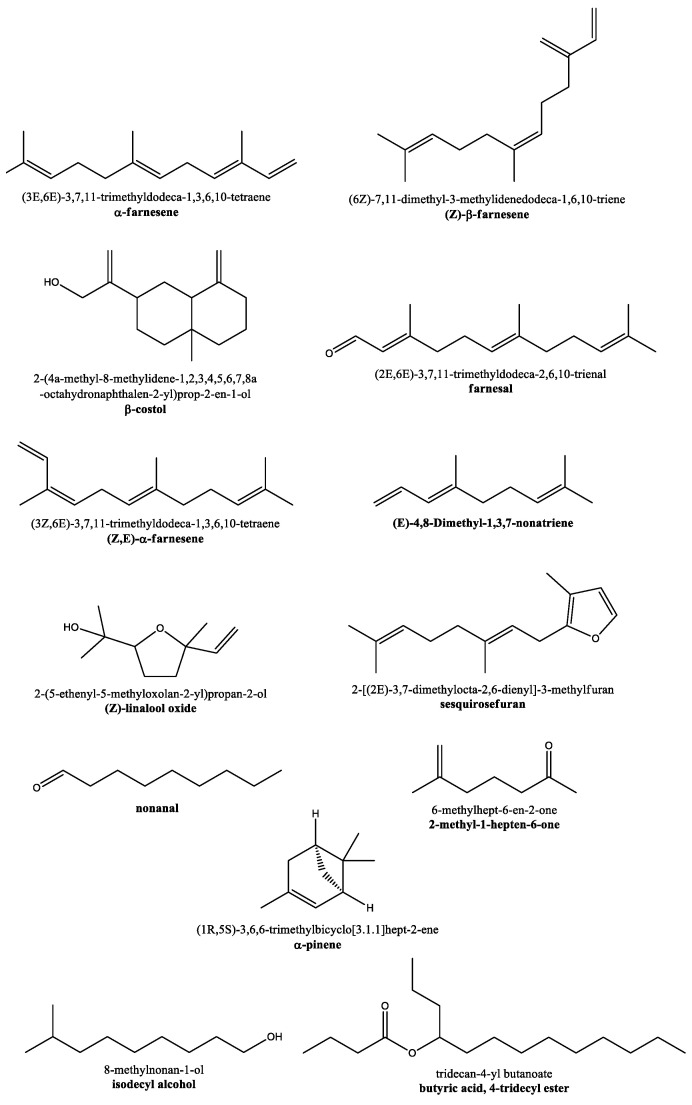
Chemical structures of some organic compounds emitted by houseplants. Note how peace lily plant emits mainly the first eight compounds (with the α-farnesene being produced at the highest levels), while the remaining five molecules (bottom) are mainly released by iron plant.

As for the volatile organic compounds emitted specifically by houseplants, the work of Yang et al. [13], based on gas chromatography–mass spectroscopy, showed that four species of popular indoor ornamental plants (such as *Spathiphyllum wallisii*, *Sansevieria trifasciata*, *Ficus benjamina*, and *Chrysalidocarpus lutescens*) were able to emit 12–23 compounds. More in detail, the lowest number of different emitted volatiles (12) was found for *Ficus benjamina*, while the highest variety (23 compounds) was observed for peace lily plant (*Spathiphyllum wallisii*, Figure 1). Interestingly, the night emanation rate was substantially lower for all house plants [13]. The highest volatile compounds emanation rate was observed into the surrounding air for peace lily plant during the daytime, with abundant releases of α-farnesene (the predominant volatile molecule), (Z)-β-farnesene, β-costol, farnesal, (Z)-linalool oxide, and others (Figure 2). *Sansevieria trifasciata*, *Ficus benjamina*, and *Chrysalidocarpus lutescens* emitted both qualitatively and quantitatively fewer volatiles than *Spathiphyllum wallisii*. Interestingly, most of the houseplant terpenoids were sesquiterpenes rather than monoterpenes [13].

Another work [29] reported an analysis of the volatile organic compounds emitted by three other common houseplants, namely, *Aspidistra elatior* (Figure 1), *Chlorophytum comosum*, and *Asparagus plumosus*, identified by thermal desorption system–gas chromatography/mass spectrum (TDS-GC/MS). Among the other indoor plants, *Aspidistra elatior* is a particularly interesting plant as it is very resistant to pests and stressful conditions of different nature, which justifies its common name, ‘iron plant’. Iron plant emitted 25 volatile organic compounds including α-pinene, aldehydes (such as nonanal), esters, and alcohols (Figure 2) [29].

Remarkably, the volatile organic compounds emitted by *Aspidistra elatior* were found to exert antimicrobial activities against *Staphylococcus aureus*, with an inhibitory rate of 38.24% [30].

### 3.2. In Silico Analysis of the Main Houseplant Volatile Organic Compounds: Vapor Pressures, Chemico-Physical/Pharmacokinetic Properties, and SARS-CoV-2 M^pro^ Inhibitory Potential Activities

Aiming at exploring some of the chemico-physical and pharmacokinetic properties of the main organic compounds emitted by indoor plants, we performed computational studies that led us to estimate the properties listed in Table 1. These include the vapor pressures, useful to compare the volatility of the compounds, the consensus partition coefficient (clogP), which gives indications on the hydrophobic nature of the molecules, the blood–brain barrier (BBB) permeability, the druglikeness (according to the Lipinski rules of five [31]), and the pan-assay interference compounds (PAINS) score, that serves to exclude for a proposed lead compound any unspecific interaction with numerous biological targets, which is clearly undesirable.

More in detail, estimating in silico the vapor pressure values at 25 °C of the indoor plant volatile organic compounds with the program UManSysProp (Table 1), we found Log vapor pressures ranging from −8.215 to −2.436, with β-costol being the less volatile of the plant-emitted compounds reported by Yang et al. [13], as we expected, given its ability to participate in H-bonds.

Interestingly, for most of the compounds we predicted favourable druglikeness properties, as well their ability to permeate the blood–brain barrier (BBB), as suggested by SwissADME for all but α-farnesene and (Z)-β-farnesene. No pan-assay interference compounds (PAINS) were found within the examined molecules, excluding, thus, that the houseplant compounds could be involved in unspecific biomolecular processes in the human body. We then performed a molecular docking analysis using the main protease of SARS-CoV-2 (PDB ID: 6Y84) as the target, and the 13 houseplant-emitted compounds (**1**–**13**, Table 1) and two reference compounds (umbelliferone, **14** and eugenol, **15**) as ligands. These latter compounds are phytochemicals taken from the literature [27] that were previously used in the molecular docking with SARS-CoV-2 M^pro^ and whose docking scores were compared with those found in our modelling to validate our methodology.

**Figure 3:**
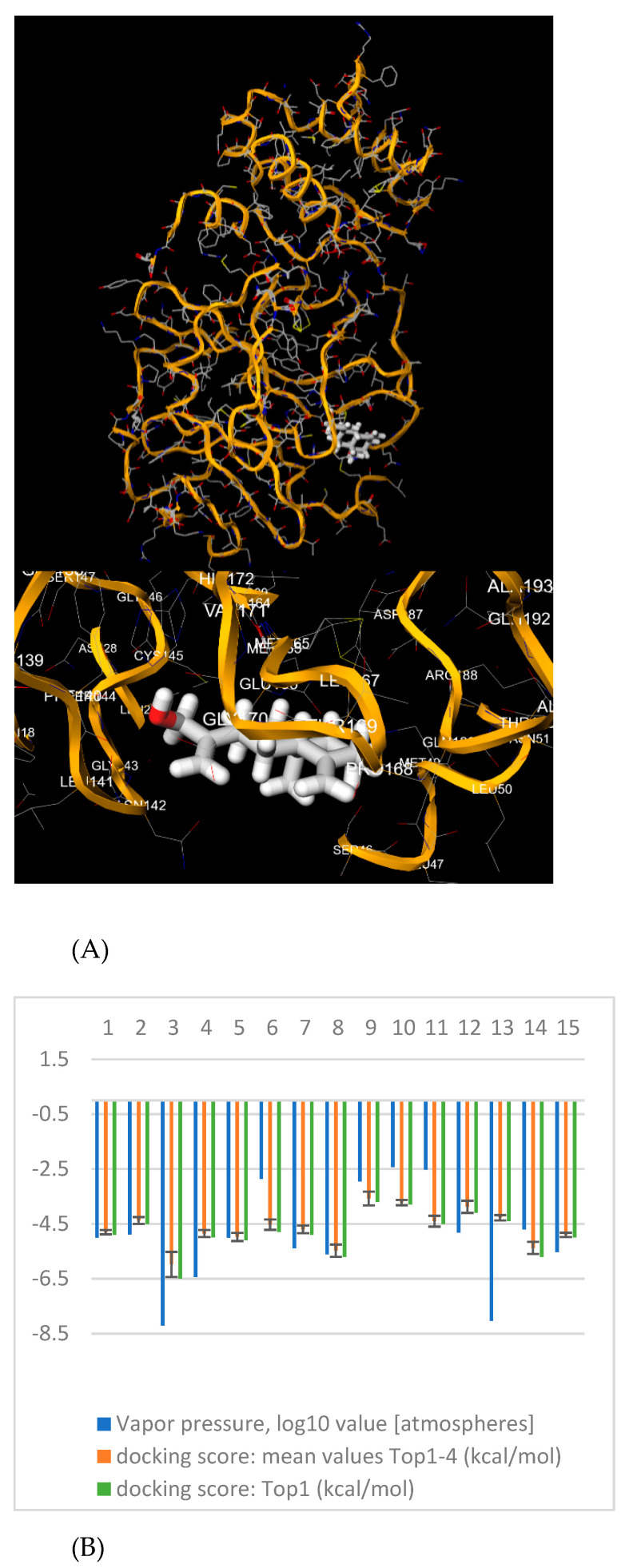
(**A**) Upper: 3D view of the top-ranked pose of the docked complex of β-costol with M^pro^ (PDB ID: 6Y84) as obtained and visualized in the 1-Click Mcule program. Bottom: Details of the binding site and the amino acids in the vicinity of β-costol (**B**) Bar graph with numerical values for the predicted vapor pressures (log10 value (atmospheres)) and docking scores (kcal/mol) for the houseplant-emitted organic compounds and reference compounds (umbelliferone and eugenol). For nomenclature of compounds (herein indicated by numbers **1**–**15**), please refer to Table 1. Note how in our computational analysis β-costol (**3**) is the less volatile compound and is also endowed with the highest affinity for the virus protease within all the phytochemicals investigated.

Our analysis revealed that β-costol (**3**) was able to form complexes with the highest affinity (with a docking score for the top-ranked pose of −6.5 kcal/mol, Table 1 and Figure 3) for the virus protease within all the organic compounds investigated, while sesquirosefuran (with a docking score for the top-ranked pose of −5.7 kcal/mol) showed an affinity comparable to the reference compound umbelliferone (−5.7 kcal/mol), and higher than eugenol (−5.0 kcal/mol), an experimentally validated inhibitor of M^pro^ [32]. Interestingly, β-costol, an oxygenated sesquiterpene particularly abundant in the *Helichrysum italicum*, a plant with antiherpesvirus properties [33], was identified also in sea cucumber (*Holothuria atra*) extracts, which similarly showed antiviral activities against both Herpes simplex virus 1 and 2 [34].

Although a predicted binding energy of −6.5 kcal/mol (found for β-costol) could not seem indicative of a good inhibitor, the following points should be considered: (1) a binding energy affinity/docking score of  −6.0 kcal/mol is often considered as the minimum threshold for drug discovery approaches based on the molecular docking with M^pro^ [35,36]; (2) since Autodock Vina software (employed in 1-Click Mcule platform) tends to underestimate the binding affinity of a ligand for its target [37], our complex could be endowed with a lower binding energy than that computed; (3) the anti-COVID-19 effects of houseplant biogenic VOCs could be synergistic, as proposed previously in the literature, for the volatile compounds isolated from the Asian evergreen plant *Melaleuca cajuputi*, which acted as synergistic M^pro^ inhibitors [38]; (4) the reference compound eugenol showed in silico an affinity score for M^pro^ even lower (Table 1) [27] than our lead compound, but in vitro it was found significantly effective in the inhibition of the protease [32].

**Figure 4:**
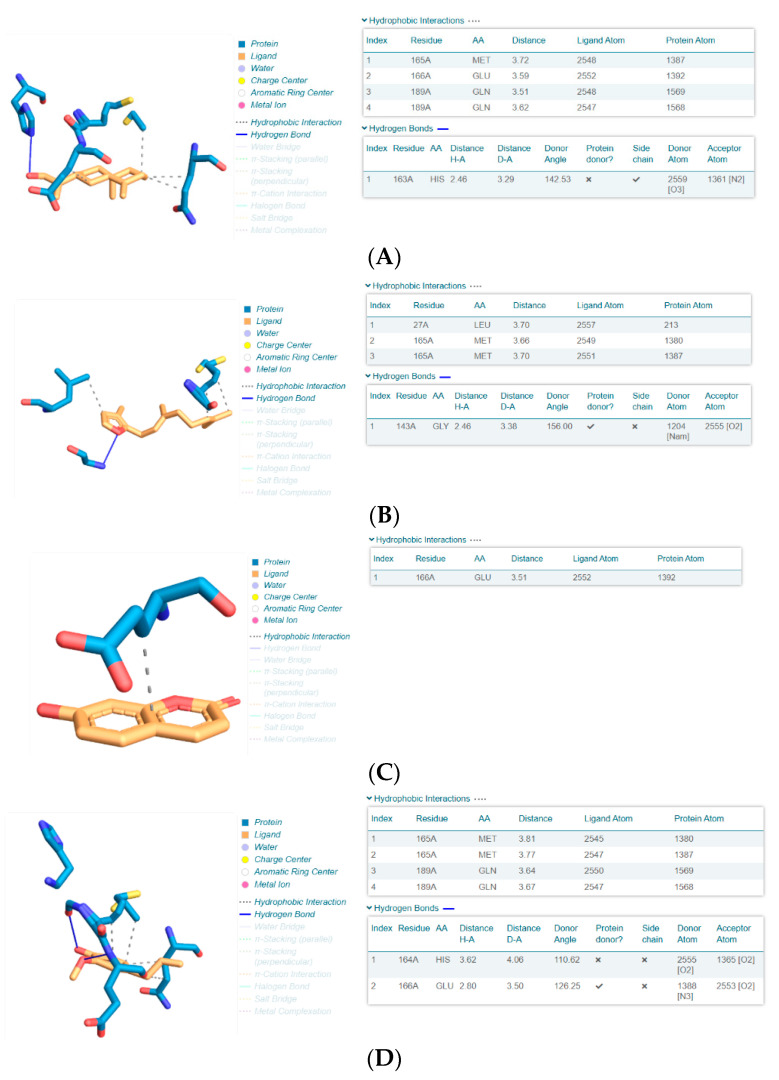
3D interactions models for the complexes of **3** (β-costol: **A**) and **8** (sesquirosefuran: **B**) as well reference compounds **14** (umbelliferone: **C**) and **15** (eugenol: **D**) obtained by PLIP software (Protein–Ligand Interaction Profiler, https://plip-tool.biotec.tu-dresden.de/, accessed on 17 November 2021). Note how **3** interacts with M^pro^ by means of a similar hydrophobic interaction as reference **14** (involving the protein residue GLU166) and **15** (MET165).

As for the interacting amino acids involved in the protease/ligand interaction, we found by complex structure analysis with PLIP software (Figure 4) that **3** interacts with M^pro^ by means of hydrophobic interactions, similar to reference compound **14**, involving i.e., the protein residue glutamic acid 166 (GLU166), and also to **15**, involving the residue methionine 165 (MET165, Figure 4), this latter interaction being observed also in the case of the complex of compound **8** with M^pro^.

Interestingly, analyzing several essential oils obtained from different plant species, it was previously found in vitro that α-pinene (emitted also by houseplants as indicated in Table 1) was associated to inhibition of SARS-CoV-1 replication [39]. On the other hand, numerous in silico studies indicated several plant BVOCs (including those emitted by houseplants, such as α-pinene, α-farnesene, and β-farnesene) as anti-COVID-19 compounds [40,41], and for some of them the anti-coronavirus activity was also proven in vitro [39].

Overall, our computational findings and the predicted druggability of the indoor plant-emitted volatile organic compounds all suggest that spending time under the canopy of plants to boost the immune system, a practice known over the globe as ‘forest bathing’ with scientifically proven benefits in the fight against viral diseases [42], could be applied also indoors in an innovative ‘indoor forest bathing’ approach. We also hypothesize the utility of nasal sprays based on mixtures of these houseplant-emitted molecules to be used for preventing COVID-19. In fact, our computational study suggest that houseplant-released volatile organic compounds and aerosol constituents could protect the human body also from the neurological complications of SARS-CoV-2 infection involving the BBB [43], thanks to both their predicted BBB permeability and potential SARS-CoV-2 M^pro^ inhibitory activity.

## 4. Conclusions

In conclusion, we explored theoretically the possibility to obtain health benefits from houseplants thanks to the biogenic compounds emitted by some of the most common species present in our homes, especially during the daytime. Through a literature analysis and subsequent in silico studies, we selected the main known compounds emitted by *Spathiphyllum wallisii* and *Aspidistra eliator*. By using molecular docking and other specific in silico methodologies utilized for vapor pressure and chemico-physical/pharmacokinetic properties prediction, we found that β-costol is an organic compound emitted in appreciable amounts by the houseplant *Spathiphyllum wallisii*, endowed with potential antiviral properties, as emerged by our MD calculations in SARS-CoV-2 M^pro^ inhibition studies, together with sesquirosefuran. Interestingly, both compounds showed comparable or higher affinities for the protease with respect to eugenol, a reference compound that was found able to hamper in vitro the enzymatic activity of M^pro^, with an inhibition constant in the sub-micromolar range [32]. Overall, our studies suggest that the anti-COVID-19 potential of some houseplant-emitted volatile compounds, coupled with their general benefits, would help sustain the utility of indoor houseplants as biogenic volatile organic compounds emitters for boosting immunity and health protection, which can thus be exploited in ‘indoor forest bathing’ approaches, that we propose not only for private houses but also public spaces, such as offices, hospitals, and schools.

## Figures and Tables

**Table 1:** Predicted properties for houseplant-emitted organic compounds and the reference compounds (umbelliferone and eugenol). All properties, where not differently indicated, were estimated by SwissADME (http://www.swissadme.ch/index.php, accessed on 17 November 2021). SMILES: simplified molecular input line entry system; BBB: blood–brain barrier; PAINS: pan-assay interference compounds; cLogP: consensus partition coefficient; S.D.: standard deviation.

Comp.	SMILES	Vapor Pressure, log_10_ at 25 °C *	cLogP	BBB Perm.	Druglikeness (Lipinski—n. Violations)	PAINS	Docking Score: Top-1 Ranked Pose (kcal/mol)	Docking Score: Mean Value ± S.D. Top 1–4 Poses (kcal/mol)
α-farnesene (**1**)	CC(=CCC/C(=C/C/C=C(\C)/C=C)/C)C	−5.008	4.96	N	Y (1)	N	−4.9	−4.80 ± 0.08
(Z)-β-farnesene (**2**)	CC(=CCC/C(=C\CCC(=C)C=C)/C)C	−4.885	4.97	N	Y (1)	N	−4.5	−4.38 ± 0.13
β-costol (**3**)	CC12CCCC(=C)C1CC(CC2)C(=C)CO	−8.215	3.66	Y	Y (0)	N	−6.5	−5.98 ± 0.46
farnesal (**4**)	CC(=CCC/C(=C/CC/C(=C/C=O)/C)/C)C	−6.437	3.66	Y	Y (0)	N	−5.0	−4.85 ± 0.13
(Z,E)-α-farnesene (**5**)	CC(=CCC/C(=C/C/C=C(/C)\C=C)/C)C	−5.008	3.66	Y	Y (0)	N	−5.1	−4.98 ± 0.15
(E)-4,8-dimethyl-1,3,7-nonatriene (**6**)	CC(=CCC/C(=C/C=C)/C)C	−2.864	3.75	Y	Y (0)	N	−4.8	−4.52 ± 0.19
(Z)-linalool oxide (**7**)	C[C@]1(CC[C@H](O1)C(C)(C)O)C=C	−5.389	2.05	Y	Y (0)	N	−4.9	−4.70 ± 0.14
Sesquirosefuran (**8**)	CC1=C(OC=C1)C/C=C(\C)/CCC=C(C)C	−5.610	4.36	Y	Y (0)	N	−5.7	−5.48 ± 0.22
Nonanal (**9**)	CCCCCCCCC=O	−2.957	2.78	Y	Y (0)	N	−3.7	−3.58 ± 0.25
2-methyl-1-hepten-6-one (**10**)	CC(=C)CCCC(=O)C	−2.436	2.11	Y	Y (0)	N	−3.8	−3.73 ± 0.10
α-pinene (**11**)	CC1=C[C@H]2C[C@@H](C1)C2(C)C	−2.523	3.06	Y	Y (1)	N	−4.5	−4.40 ± 0.20
isodecyl alcohol (**12**)	CC(C)CCCCCCCO	−4.821	3.44	Y	Y (0)	N	−4.1	−3.88 ± 0.22
butyric acid, 4-tridecyl ester (**13**)	CCCCCCCCCCCC(C)OC(=O)CCC	−8.032	5.42	Y	Y (1)	N	−4.4	−4.28 ± 0.10
umbelliferone (**14**)	C1=CC(=CC2=C1C=CC(=O)O2)O	−4.705	1.51	Y	Y (0)	N	−5.7	−5.38 ± 0.22
eugenol (**15**)	COC1=C(C=CC(=C1)CC=C)O	−5.531	2.25	Y	Y (0)	N	−5.0	−4.90 ± 0.08

* Calculated by UManSysProp (http://umansysprop.seaes.manchester.ac.uk/tool/vapour_pressure, accessed on 17 November 2021); ‘Nannoolal 2008’ vapor pressure method; ‘Joback and Reid 1987’ boiling point method, at 298.15 K (vapor pressure as log_10_ value (atmospheres)).

## Supplementary Materials

The following are available online at https://www.mdpi.com/article/10.3390/ijerph19010273/s1, Figures S1–S15: The chemico-physical and pharmacokinetic properties predictions (performed by SwissADME online program, http://www.swissadme.ch/index.php, accessed on 17 November 2021) for compounds **1**–**15** are reported.
