# Supplementary material for: Evaluating In Silico the Potential Health and Environmental Benefits of Houseplant Volatile Organic Compounds for an Emerging ‘Indoor Forest Bathing’ Approach
Source: Int J Environ Res Public Health. 2021 Dec 27;19(1):273. doi: 10.3390/ijerph19010273 (PMC8751036; doi:10.3390/ijerph19010273)
Supplement: Supplementary file 1 [file ijerph-19-00273-s001.zip › ijerph-1492886-supplementary.pdf]

Supporting Information

Evaluating *in silico* the Potential Health and Environmental Benefits of Houseplant Volatile organic Compounds for an Emerging 'Indoor Forest Bathing' Approach

Valentina Roviello [1,*], Pasqualina Liana Scognamiglio [2], Ugo Caruso [3], Caterina Vicidomini [4], and Giovanni N. Roviello [4,*]

1 Department of Chemical, Materials and Industrial Production Engineering (DICMaPI), University of Naples Federico II, Piazzale V. Tecchio 80, 80125 Naples, Italy; valentina.roviello@unina.it
2 Center for Advanced Biomaterial for Health Care (CABHC), Istituto Italiano di Tecnologia, I-80125 Naples, Italy; pasqualina.scognamiglio@iit.it
3 Department of Chemical Sciences, University of Naples Federico II, Via Cintia 21, 80126 Naples, Italy; ugo.caruso@unina.it
4 Istituto di Biostrutture e Bioimmagini IBB-CNR; via Tommaso De Amicis 95, I-80145 Naples, Italy; giroviel@unina.it

The chemico-physical and pharmacokinetic properties predictions (performed by SwissADME online program, http://www.swissadme.ch/index.php accessed on 17th November 2021) for compounds 1–15 (Table 1) are reported below.

α-farnesene

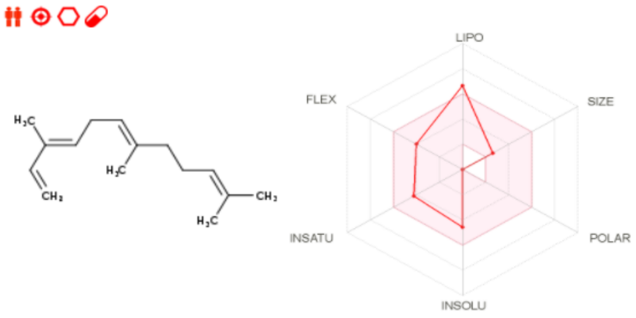

SMILES C=C/C(=C/C/C=C(/CCC=C(C)C)\C)/C

Physicochemical Properties	
Formula	C15H24
Molecular weight	204.35 g/mol
Num. heavy atoms	15
Num. arom. heavy atoms	0
Fraction Csp3	0.47
Num. rotatable bonds	6
Num. H-bond acceptors	0
Num. H-bond donors	0
Molar Refractivity	72.32
TPSA	0.00 Å²

Lipophilicity	
Log $P_{o/w}$ (iLOGP)	3.89
Log $P_{o/w}$ (XLOGP3)	6.12
Log $P_{o/w}$ (WLOGP)	5.20
Log $P_{o/w}$ (MLOGP)	4.84
Log $P_{o/w}$ (SILICOS-IT)	4.76
Consensus Log $P_{o/w}$	4.96

Water Solubility	
Log *S* (ESOL)	-4.57
Solubility	5.54e-03 mg/ml ; 2.71e-05 mol/l
Class	Moderately soluble
Log *S* (Ali)	-5.90
Solubility	2.56e-04 mg/ml ; 1.25e-06 mol/l
Class	Moderately soluble
Log *S* (SILICOS-IT)	-3.37
Solubility	8.82e-02 mg/ml ; 4.32e-04 mol/l
Class	Soluble

Pharmacokinetics	
GI absorption	Low
BBB permeant	No
P-gp substrate	No
CYP1A2 inhibitor	Yes
CYP2C19 inhibitor	No
CYP2C9 inhibitor	Yes
CYP2D6 inhibitor	No
CYP3A4 inhibitor	No
Log K_p (skin permeation)	-3.20 cm/s

Druglikeness	
Lipinski	Yes; 1 violation: MLOGP>4.15
Ghose	Yes
Veber	Yes
Egan	Yes
Muegge	No; 2 violations: XLOGP3>5, Heteroatoms<2
Bioavailability Score	0.55

Medicinal Chemistry	
PAINS	0 alert
Brenk	2 alerts: isolated_alkene, polyene
Leadlikeness	No; 2 violations: MW<250, XLOGP3>3.5
Synthetic accessibility	3.72

Figure S1 Chemico-physical and pharmacokinetic properties predicted for α-farnesene.

(Z)-β -farnesene

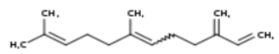

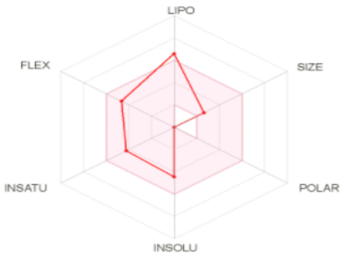

SMILES C=CC(=C)CC/C=C(\CCC=C(C)C)/C

Physicochemical Properties	
Formula	C15H24
Molecular weight	204.35 g/mol
Num. heavy atoms	15
Num. arom. heavy atoms	0
Fraction Csp3	0.47
Num. rotatable bonds	7
Num. H-bond acceptors	0
Num. H-bond donors	0
Molar Refractivity	72.32
TPSA	0.00 Å²
**Lipophilicity**	
Log $P_{o/w}$ (iLOGP)	3.86
Log $P_{o/w}$ (XLOGP3)	6.03
Log $P_{o/w}$ (WLOGP)	5.20
Log $P_{o/w}$ (MLOGP)	4.84
Log $P_{o/w}$ (SILICOS-IT)	4.93
Consensus Log $P_{o/w}$	4.97

Water Solubility	
Log *S* (ESOL)	-4.44
Solubility	7.35e-03 mg/ml ; 3.60e-05 mol/l
Class	Moderately soluble
Log *S* (Ali)	-5.81
Solubility	3.18e-04 mg/ml ; 1.55e-06 mol/l
Class	Moderately soluble
Log *S* (SILICOS-IT)	-3.74
Solubility	3.73e-02 mg/ml ; 1.82e-04 mol/l
Class	Soluble
**Pharmacokinetics**	
GI absorption	Low
BBB permeant	No
P-gp substrate	No
CYP1A2 inhibitor	Yes
CYP2C19 inhibitor	No
CYP2C9 inhibitor	Yes
CYP2D6 inhibitor	No
CYP3A4 inhibitor	No
Log K_p (skin permeation)	-3.27 cm/s
**Druglikeness**	
Lipinski	Yes; 1 violation: MLOGP>4.15
Ghose	Yes
Veber	Yes
Egan	Yes
Muegge	No; 2 violations: XLOGP3>5, Heteroatoms<2
Bioavailability Score	0.55
**Medicinal Chemistry**	
PAINS	0 alert
Brenk	2 alerts: isolated_alkene, polyene
Leadlikeness	No; 2 violations: MW<250, XLOGP3>3.5
Synthetic accessibility	3.42

Figure S2 Chemico-physical and pharmacokinetic properties predicted for (Z)-β-farnesene.

β-costol

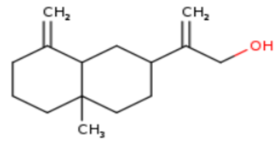

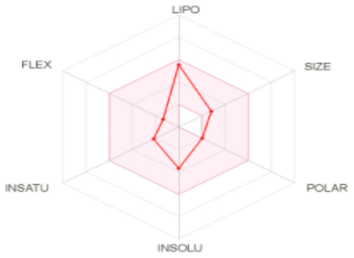

SMILES OCC(=C)C1CCC2(C(C1)C(=C)CCC2)C

Physicochemical Properties	
Formula	C15H24O
Molecular weight	220.35 g/mol
Num. heavy atoms	16
Num. arom. heavy atoms	0
Fraction Csp3	0.73
Num. rotatable bonds	2
Num. H-bond acceptors	1
Num. H-bond donors	1
Molar Refractivity	69.94
TPSA	20.23 Å²
**Lipophilicity**	
Log $P_{o/w}$ (iLOGP)	3.05
Log $P_{o/w}$ (XLOGP3)	4.19
Log $P_{o/w}$ (WLOGP)	3.70
Log $P_{o/w}$ (MLOGP)	3.56
Log $P_{o/w}$ (SILICOS-IT)	3.80
Consensus Log $P_{o/w}$	3.66

Water Solubility	
Log *S* (ESOL)	-3.71
Solubility	4.26e-02 mg/ml ; 1.93e-04 mol/l
Class	Soluble
Log *S* (Ali)	-4.32
Solubility	1.05e-02 mg/ml ; 4.74e-05 mol/l
Class	Moderately soluble
Log *S* (SILICOS-IT)	-3.24
Solubility	1.27e-01 mg/ml ; 5.74e-04 mol/l
Class	Soluble
**Pharmacokinetics**	
GI absorption	High
BBB permeant	Yes
P-gp substrate	No
CYP1A2 inhibitor	No
CYP2C19 inhibitor	Yes
CYP2C9 inhibitor	Yes
CYP2D6 inhibitor	No
CYP3A4 inhibitor	No
Log K_p (skin permeation)	-4.67 cm/s
**Druglikeness**	
Lipinski	Yes; 0 violation
Ghose	Yes
Veber	Yes
Egan	Yes
Muegge	No; 1 violation: Heteroatoms<2
Bioavailability Score	0.55
**Medicinal Chemistry**	
PAINS	0 alert
Brenk	1 alert: isolated_alkene
Leadlikeness	No; 2 violations: MW<250, XLOGP3>3.5
Synthetic accessibility	3.28

Figure S3 Chemico-physical and pharmacokinetic properties predicted for β-costol.

farnesal

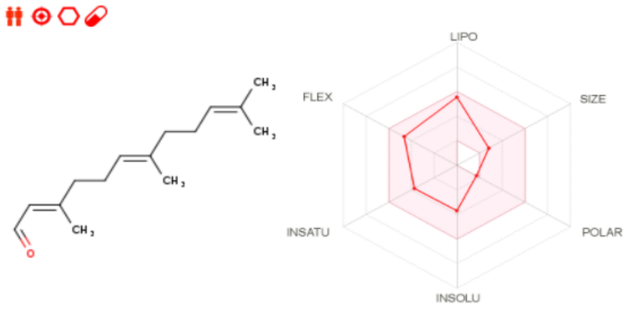

SMILES O=C/C=C(/CC/C=C(/CCC=C(C)C)\C)\C

Physicochemical Properties	
Formula	C15H24O
Molecular weight	220.35 g/mol
Num. heavy atoms	16
Num. arom. heavy atoms	0
Fraction Csp3	0.53
Num. rotatable bonds	7
Num. H-bond acceptors	1
Num. H-bond donors	0
Molar Refractivity	73.00
TPSA	17.07 Å²
**Lipophilicity**	
Log $P_{o/w}$ (iLOGP)	3.05
Log $P_{o/w}$ (XLOGP3)	4.19
Log $P_{o/w}$ (WLOGP)	4.60
Log $P_{o/w}$ (MLOGP)	3.56
Log $P_{o/w}$ (SILICOS-IT)	3.80
Consensus Log $P_{o/w}$	3.66

Water Solubility	
Log *S* (ESOL)	-3.71
Solubility	4.26e-02 mg/ml ; 1.93e-04 mol/l
Class	Soluble
Log *S* (Ali)	-4.32
Solubility	1.05e-02 mg/ml ; 4.74e-05 mol/l
Class	Moderately soluble
Log *S* (SILICOS-IT)	-3.24
Solubility	1.27e-01 mg/ml ; 5.74e-04 mol/l
Class	Soluble
**Pharmacokinetics**	
GI absorption	High
BBB permeant	Yes
P-gp substrate	No
CYP1A2 inhibitor	No
CYP2C19 inhibitor	Yes
CYP2C9 inhibitor	Yes
CYP2D6 inhibitor	No
CYP3A4 inhibitor	No
Log K_p (skin permeation)	-4.67 cm/s
**Druglikeness**	
Lipinski	Yes; 0 violation
Ghose	Yes
Veber	Yes
Egan	Yes
Muegge	No; 1 violation: Heteroatoms<2
Bioavailability Score	0.55
**Medicinal Chemistry**	
PAINS	0 alert
Brenk	1 alert: isolated_alkene
Leadlikeness	No; 2 violations: MW<250, XLOGP3>3.5
Synthetic accessibility	3.28

Figure S4 Chemico-physical and pharmacokinetic properties predicted for farnesal.

(Z,E)-α-farnesene

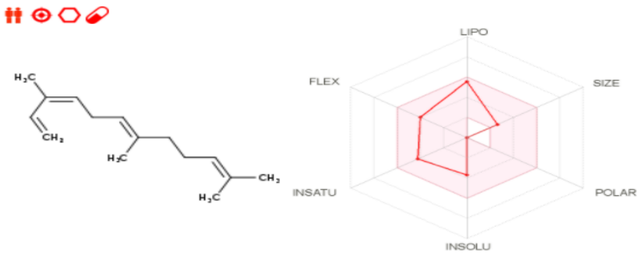

SMILES C=C/C(=C\C/C=C(/CCC=C(C)C)\C)/C

Physicochemical Properties	
Formula	C15H24
Molecular weight	204.35 g/mol
Num. heavy atoms	15
Num. arom. heavy atoms	0
Fraction Csp3	0.47
Num. rotatable bonds	6
Num. H-bond acceptors	0
Num. H-bond donors	0
Molar Refractivity	72.32
TPSA	0.00 Å²
**Lipophilicity**	
Log $P_{o/w}$ (iLOGP)	3.05
Log $P_{o/w}$ (XLOGP3)	4.19
Log $P_{o/w}$ (WLOGP)	5.20
Log $P_{o/w}$ (MLOGP)	3.56
Log $P_{o/w}$ (SILICOS-IT)	3.80
Consensus Log $P_{o/w}$	3.66

Water Solubility	
Log *S* (ESOL)	-3.71
Solubility	4.26e-02 mg/ml ; 1.93e-04 mol/l
Class	Soluble
Log *S* (Ali)	-4.32
Solubility	1.05e-02 mg/ml ; 4.74e-05 mol/l
Class	Moderately soluble
Log *S* (SILICOS-IT)	-3.24
Solubility	1.27e-01 mg/ml ; 5.74e-04 mol/l
Class	Soluble
**Pharmacokinetics**	
GI absorption	High
BBB permeant	Yes
P-gp substrate	No
CYP1A2 inhibitor	No
CYP2C19 inhibitor	Yes
CYP2C9 inhibitor	Yes
CYP2D6 inhibitor	No
CYP3A4 inhibitor	No
Log K_p (skin permeation)	-4.67 cm/s
**Druglikeness**	
Lipinski	Yes; 0 violation
Ghose	Yes
Veber	Yes
Egan	Yes
Muegge	No; 1 violation: Heteroatoms<2
Bioavailability Score	0.55
**Medicinal Chemistry**	
PAINS	0 alert
Brenk	1 alert: isolated_alkene
Leadlikeness	No; 2 violations: MW<250, XLOGP3>3.5
Synthetic accessibility	3.28

Figure S5 Chemico-physical and pharmacokinetic properties predicted for (Z,E)-α-farnesene.

(E)-4,8-dimethyl-1,3,7-nonatriene

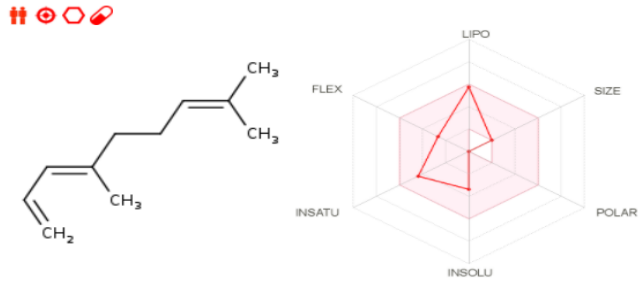

SMILES C=C/C=C(/CCC=C(C)C)\C

Physicochemical Properties	
Formula	C11H18
Molecular weight	150.26 g/mol
Num. heavy atoms	11
Num. arom. heavy atoms	0
Fraction Csp3	0.45
Num. rotatable bonds	4
Num. H-bond acceptors	0
Num. H-bond donors	0
Molar Refractivity	53.57
TPSA	0.00 Å²
**Lipophilicity**	
Log $P_{o/w}$ (iLOGP)	3.16
Log $P_{o/w}$ (XLOGP3)	4.56
Log $P_{o/w}$ (WLOGP)	3.87
Log $P_{o/w}$ (MLOGP)	3.85
Log $P_{o/w}$ (SILICOS-IT)	3.32
Consensus Log $P_{o/w}$	3.75

Water Solubility	
Log *S* (ESOL)	-3.38
Solubility	6.26e-02 mg/ml ; 4.16e-04 mol/l
Class	Soluble
Log *S* (Ali)	-4.28
Solubility	7.83e-03 mg/ml ; 5.21e-05 mol/l
Class	Moderately soluble
Log *S* (SILICOS-IT)	-2.46
Solubility	5.23e-01 mg/ml ; 3.48e-03 mol/l
Class	Soluble
**Pharmacokinetics**	
GI absorption	Low
BBB permeant	Yes
P-gp substrate	No
CYP1A2 inhibitor	No
CYP2C19 inhibitor	No
CYP2C9 inhibitor	No
CYP2D6 inhibitor	No
CYP3A4 inhibitor	No
Log K_p (skin permeation)	-3.98 cm/s
**Druglikeness**	
Lipinski	Yes; 0 violation
Ghose	No; 1 violation: MW<160
Veber	Yes
Egan	Yes
Muegge	No; 2 violations: MW<200, Heteroatoms<2
Bioavailability Score	0.55
**Medicinal Chemistry**	
PAINS	0 alert
Brenk	2 alerts: isolated_alkene, polyene
Leadlikeness	No; 2 violations: MW<250, XLOGP3>3.5
Synthetic accessibility	3.27

Figure S6 Chemico-physical and pharmacokinetic properties predicted for (E)-4,8-dimethyl-1,3,7-nonatriene.

(Z)-linalool oxide

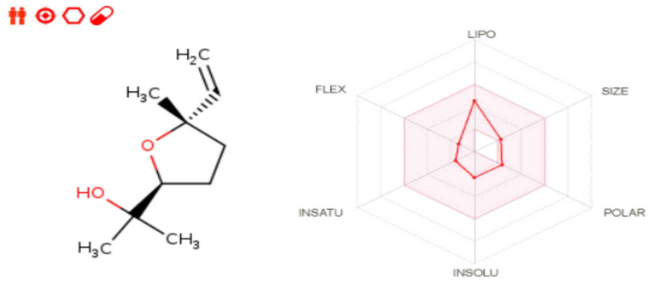

SMILES C=C[C@]1(C)CC[C@H](O1)C(O)(C)C

Physicochemical Properties	
Formula	C10H18O2
Molecular weight	170.25 g/mol
Num. heavy atoms	12
Num. arom. heavy atoms	0
Fraction Csp3	0.80
Num. rotatable bonds	2
Num. H-bond acceptors	2
Num. H-bond donors	1
Molar Refractivity	49.92
TPSA	29.46 Å²
**Lipophilicity**	
Log $P_{o/w}$ (iLOGP)	2.44
Log $P_{o/w}$ (XLOGP3)	2.43
Log $P_{o/w}$ (WLOGP)	1.88
Log $P_{o/w}$ (MLOGP)	1.38
Log $P_{o/w}$ (SILICOS-IT)	2.10
Consensus Log $P_{o/w}$	2.05

Water Solubility	
Log *S* (ESOL)	-2.29
Solubility	8.64e-01 mg/ml ; 5.08e-03 mol/l
Class	Soluble
Log *S* (Ali)	-2.69
Solubility	3.46e-01 mg/ml ; 2.03e-03 mol/l
Class	Soluble
Log *S* (SILICOS-IT)	-1.56
Solubility	4.66e+00 mg/ml ; 2.74e-02 mol/l
Class	Soluble
**Pharmacokinetics**	
GI absorption	High
BBB permeant	Yes
P-gp substrate	No
CYP1A2 inhibitor	No
CYP2C19 inhibitor	No
CYP2C9 inhibitor	No
CYP2D6 inhibitor	No
CYP3A4 inhibitor	No
Log K_p (skin permeation)	-5.61 cm/s
**Druglikeness**	
Lipinski	Yes; 0 violation
Ghose	Yes
Veber	Yes
Egan	Yes
Muegge	No; 1 violation: MW<200
Bioavailability Score	0.55
**Medicinal Chemistry**	
PAINS	0 alert
Brenk	1 alert: isolated_alkene
Leadlikeness	No; 1 violation: MW<250
Synthetic accessibility	3.20

Figure S7 Chemico-physical and pharmacokinetic properties predicted for (Z)-linalool oxide.

sesquirosefuran

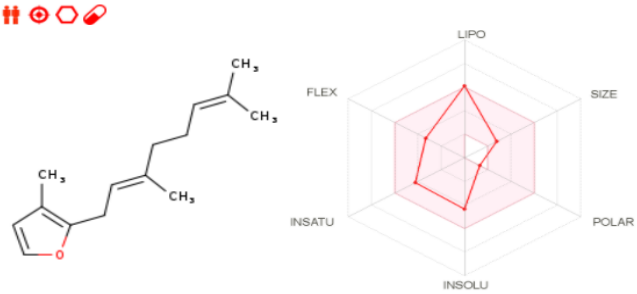

SMILES C/C(=C\Cc1occc1C)/CCC=C(C)C

Physicochemical Properties	
Formula	C15H22O
Molecular weight	218.33 g/mol
Num. heavy atoms	16
Num. arom. heavy atoms	5
Fraction Csp3	0.47
Num. rotatable bonds	5
Num. H-bond acceptors	1
Num. H-bond donors	0
Molar Refractivity	70.95
TPSA	13.14 Å²

Lipophilicity	
Log $P_{o/w}$ (iLOGP)	3.68
Log $P_{o/w}$ (XLOGP3)	5.17
Log $P_{o/w}$ (WLOGP)	4.82
Log $P_{o/w}$ (MLOGP)	3.33
Log $P_{o/w}$ (SILICOS-IT)	4.78
Consensus Log $P_{o/w}$	4.36

Water Solubility	
Log *S* (ESOL)	-4.35
Solubility	9.71e-03 mg/ml ; 4.45e-05 mol/l
Class	Moderately soluble
Log *S* (Ali)	-5.19
Solubility	1.40e-03 mg/ml ; 6.43e-06 mol/l
Class	Moderately soluble
Log *S* (SILICOS-IT)	-4.56
Solubility	6.00e-03 mg/ml ; 2.75e-05 mol/l
Class	Moderately soluble

Pharmacokinetics	
GI absorption	High
BBB permeant	Yes
P-gp substrate	No
CYP1A2 inhibitor	Yes
CYP2C19 inhibitor	Yes
CYP2C9 inhibitor	No
CYP2D6 inhibitor	No
CYP3A4 inhibitor	No
Log K_p (skin permeation)	-3.96 cm/s

Druglikeness	
Lipinski	Yes; 0 violation
Ghose	Yes
Veber	Yes
Egan	Yes
Muegge	No; 2 violations: XLOGP3>5, Heteroatoms<2
Bioavailability Score	0.55

Medicinal Chemistry	
PAINS	0 alert
Brenk	1 alert: isolated_alkene
Leadlikeness	No; 2 violations: MW<250, XLOGP3>3.5
Synthetic accessibility	3.45

Figure S8 Chemico-physical and pharmacokinetic properties predicted for sesquirosefuran.

nonanal

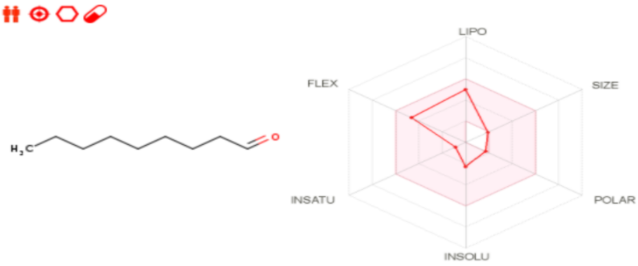

SMILES CCCCCCCCC=O

Physicochemical Properties	
Formula	C9H18O
Molecular weight	142.24 g/mol
Num. heavy atoms	10
Num. arom. heavy atoms	0
Fraction Csp3	0.89
Num. rotatable bonds	7
Num. H-bond acceptors	1
Num. H-bond donors	0
Molar Refractivity	45.58
TPSA	17.07 Å²

Lipophilicity	
Log $P_{o/w}$ (iLOGP)	2.44
Log $P_{o/w}$ (XLOGP3)	3.26
Log $P_{o/w}$ (WLOGP)	2.94
Log $P_{o/w}$ (MLOGP)	2.39
Log $P_{o/w}$ (SILICOS-IT)	2.88
Consensus Log $P_{o/w}$	2.78

Water Solubility	
Log *S* (ESOL)	-2.31
Solubility	6.91e-01 mg/ml ; 4.86e-03 mol/l
Class	Soluble
Log *S* (Ali)	-3.29
Solubility	7.25e-02 mg/ml ; 5.10e-04 mol/l
Class	Soluble
Log *S* (SILICOS-IT)	-3.02
Solubility	1.35e-01 mg/ml ; 9.46e-04 mol/l
Class	Soluble

Pharmacokinetics	
GI absorption	High
BBB permeant	Yes
P-gp substrate	No
CYP1A2 inhibitor	No
CYP2C19 inhibitor	No
CYP2C9 inhibitor	No
CYP2D6 inhibitor	No
CYP3A4 inhibitor	No
Log K_p (skin permeation)	-4.85 cm/s

Druglikeness	
Lipinski	Yes; 0 violation
Ghose	No; 1 violation: MW<160
Veber	Yes
Egan	Yes
Muegge	No; 2 violations: MW<200, Heteroatoms<2
Bioavailability Score	0.55

Medicinal Chemistry	
PAINS	0 alert
Brenk	1 alert: aldehyde
Leadlikeness	No; 1 violation: MW<250
Synthetic accessibility	1.52

Figure S9 Chemico-physical and pharmacokinetic properties predicted for nonanal.

2-methyl-1-hepten-6-one

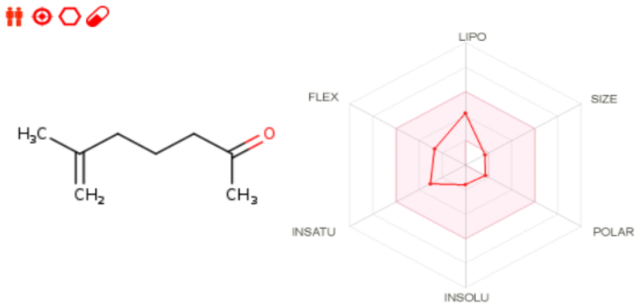

SMILES CC(=C)CCCC(=O)C

Physicochemical Properties	
Formula	C8H14O
Molecular weight	126.20 g/mol
Num. heavy atoms	9
Num. arom. heavy atoms	0
Fraction Csp3	0.62
Num. rotatable bonds	4
Num. H-bond acceptors	1
Num. H-bond donors	0
Molar Refractivity	40.30
TPSA	17.07 Å²

Lipophilicity	
Log $P_{o/w}$ (iLOGP)	2.14
Log $P_{o/w}$ (XLOGP3)	1.97
Log $P_{o/w}$ (WLOGP)	2.32
Log $P_{o/w}$ (MLOGP)	1.97
Log $P_{o/w}$ (SILICOS-IT)	2.13
Consensus Log $P_{o/w}$	2.11

Water Solubility	
Log *S* (ESOL)	-1.60
Solubility	3.17e+00 mg/ml ; 2.51e-02 mol/l
Class	Very soluble
Log *S* (Ali)	-1.95
Solubility	1.40e+00 mg/ml ; 1.11e-02 mol/l
Class	Very soluble
Log *S* (SILICOS-IT)	-2.22
Solubility	7.57e-01 mg/ml ; 6.00e-03 mol/l
Class	Soluble

Pharmacokinetics	
GI absorption	High
BBB permeant	Yes
P-gp substrate	No
CYP1A2 inhibitor	No
CYP2C19 inhibitor	No
CYP2C9 inhibitor	No
CYP2D6 inhibitor	No
CYP3A4 inhibitor	No
Log K_p (skin permeation)	-5.67 cm/s

Druglikeness	
Lipinski	Yes; 0 violation
Ghose	No; 1 violation: MW<160
Veber	Yes
Egan	Yes
Muegge	No; 2 violations: MW<200, Heteroatoms<2
Bioavailability Score	0.55

Medicinal Chemistry	
PAINS	0 alert
Brenk	1 alert: isolated_alkene
Leadlikeness	No; 1 violation: MW<250
Synthetic accessibility	1.80

Figure S10 Chemico-physical and pharmacokinetic properties predicted for 2-methyl-1-hepten-6-one.

α-pinene

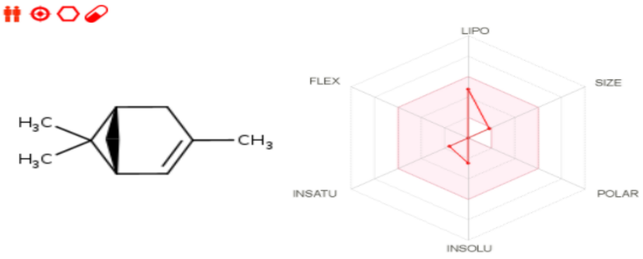

SMILES CC1=C[C@H]2C[C@@H](C1)C2(C)C

Physicochemical Properties	
Formula	C10H16
Molecular weight	136.23 g/mol
Num. heavy atoms	10
Num. arom. heavy atoms	0
Fraction Csp3	0.80
Num. rotatable bonds	0
Num. H-bond acceptors	0
Num. H-bond donors	0
Molar Refractivity	45.22
TPSA	0.00 Å²

Lipophilicity	
Log $P_{o/w}$ (iLOGP)	2.63
Log $P_{o/w}$ (XLOGP3)	2.85
Log $P_{o/w}$ (WLOGP)	3.00
Log $P_{o/w}$ (MLOGP)	4.29
Log $P_{o/w}$ (SILICOS-IT)	2.79
Consensus Log $P_{o/w}$	3.11

Water Solubility	
Log *S* (ESOL)	-2.48
Solubility	4.51e-01 mg/ml ; 3.31e-03 mol/l
Class	Soluble
Log *S* (Ali)	-2.51
Solubility	4.22e-01 mg/ml ; 3.10e-03 mol/l
Class	Soluble
Log *S* (SILICOS-IT)	-2.23
Solubility	8.06e-01 mg/ml ; 5.92e-03 mol/l
Class	Soluble

Pharmacokinetics	
GI absorption	Low
BBB permeant	Yes
P-gp substrate	No
CYP1A2 inhibitor	No
CYP2C19 inhibitor	No
CYP2C9 inhibitor	No
CYP2D6 inhibitor	No
CYP3A4 inhibitor	No
Log K_p (skin permeation)	-5.11 cm/s

Druglikeness	
Lipinski	Yes; 1 violation: MLOGP>4.15
Ghose	No; 1 violation: MW<160
Veber	Yes
Egan	Yes
Muegge	No; 2 violations: MW<200, Heteroatoms<2
Bioavailability Score	0.55

Medicinal Chemistry	
PAINS	0 alert
Brenk	1 alert: isolated_alkene
Leadlikeness	No; 1 violation: MW<250
Synthetic accessibility	4.44

Figure S11 Chemico-physical and pharmacokinetic properties predicted for α-pinene.

isodecyl alcohol

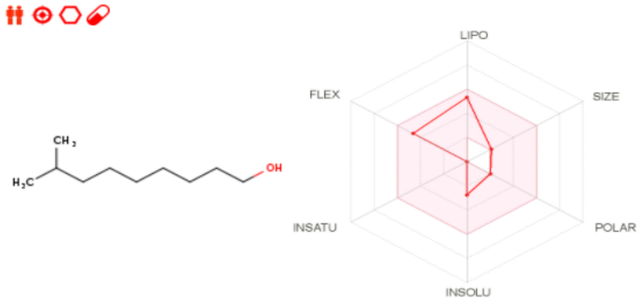

SMILES OCCCCCCCC(C)C

Physicochemical Properties	
Formula	C10H22O
Molecular weight	158.28 g/mol
Num. heavy atoms	11
Num. arom. heavy atoms	0
Fraction Csp3	1.00
Num. rotatable bonds	7
Num. H-bond acceptors	1
Num. H-bond donors	1
Molar Refractivity	51.35
TPSA	20.23 Å²

Lipophilicity	
Log $P_{o/w}$ (iLOGP)	2.84
Log $P_{o/w}$ (XLOGP3)	3.79
Log $P_{o/w}$ (WLOGP)	2.98
Log $P_{o/w}$ (MLOGP)	2.84
Log $P_{o/w}$ (SILICOS-IT)	2.84
Consensus Log $P_{o/w}$	3.06

Water Solubility	
Log *S* (ESOL)	-2.75
Solubility	2.83e-01 mg/ml ; 1.79e-03 mol/l
Class	Soluble
Log *S* (Ali)	-3.91
Solubility	1.95e-02 mg/ml ; 1.23e-04 mol/l
Class	Soluble
Log *S* (SILICOS-IT)	-2.95
Solubility	1.78e-01 mg/ml ; 1.13e-03 mol/l
Class	Soluble

Pharmacokinetics	
GI absorption	High
BBB permeant	Yes
P-gp substrate	No
CYP1A2 inhibitor	No
CYP2C19 inhibitor	No
CYP2C9 inhibitor	No
CYP2D6 inhibitor	No
CYP3A4 inhibitor	No
Log K_p (skin permeation)	-4.57 cm/s

Druglikeness	
Lipinski	Yes; 0 violation
Ghose	No; 1 violation: MW<160
Veber	Yes
Egan	Yes
Muegge	No; 2 violations: MW<200, Heteroatoms<2
Bioavailability Score	0.55

Medicinal Chemistry	
PAINS	0 alert
Brenk	0 alert
Leadlikeness	No; 2 violations: MW<250, XLOGP3>3.5
Synthetic accessibility	1.64

Figure S12 Chemico-physical and pharmacokinetic properties predicted for isodecyl alcohol.

butyric acid, 4-tridecyl ester

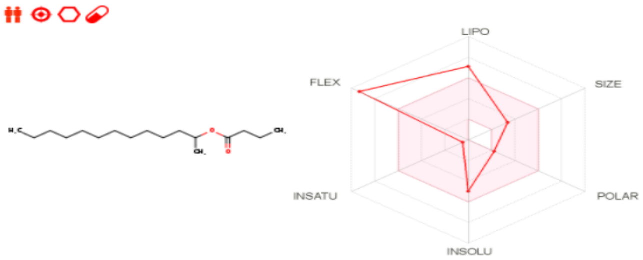

SMILES CCCCCCCCCCCC(OC(=O)CCC)C

Physicochemical Properties	
Formula	C17H34O2
Molecular weight	270.45 g/mol
Num. heavy atoms	19
Num. arom. heavy atoms	0
Fraction Csp3	0.94
Num. rotatable bonds	14
Num. H-bond acceptors	2
Num. H-bond donors	0
Molar Refractivity	85.12
TPSA	26.30 Å²

Lipophilicity	
Log $P_{o/w}$ (iLOGP)	4.40
Log $P_{o/w}$ (XLOGP3)	6.96
Log $P_{o/w}$ (WLOGP)	5.64
Log $P_{o/w}$ (MLOGP)	4.44
Log $P_{o/w}$ (SILICOS-IT)	5.67
Consensus Log $P_{o/w}$	5.42

Water Solubility	
Log *S* (ESOL)	-4.98
Solubility	2.85e-03 mg/ml ; 1.05e-05 mol/l
Class	Moderately soluble
Log *S* (Ali)	-7.33
Solubility	1.28e-05 mg/ml ; 4.72e-08 mol/l
Class	Poorly soluble
Log *S* (SILICOS-IT)	-5.64
Solubility	6.24e-04 mg/ml ; 2.31e-06 mol/l
Class	Moderately soluble

Pharmacokinetics	
GI absorption	High
BBB permeant	Yes
P-gp substrate	No
CYP1A2 inhibitor	No
CYP2C19 inhibitor	No
CYP2C9 inhibitor	Yes
CYP2D6 inhibitor	No
CYP3A4 inhibitor	No
Log K_p (skin permeation)	-3.01 cm/s

Druglikeness	
Lipinski	Yes; 1 violation: MLOGP>4.15
Ghose	No; 1 violation: WLOGP>5.6
Veber	No; 1 violation: Rotors>10
Egan	Yes
Muegge	No; 1 violation: XLOGP3>5
Bioavailability Score	0.55

Medicinal Chemistry	
PAINS	0 alert
Brenk	0 alert
Leadlikeness	No; 2 violations: Rotors>7, XLOGP3>3.5
Synthetic accessibility	3.10

Figure S13 Chemico-physical and pharmacokinetic properties predicted for butyric acid, 4-tridecyl ester.

umbelliferone

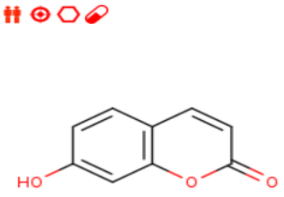

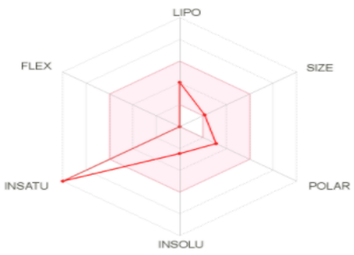

SMILES Oc1ccc2c(c1)oc(=O)cc2

Physicochemical Properties	
Formula	C9H6O3
Molecular weight	162.14 g/mol
Num. heavy atoms	12
Num. arom. heavy atoms	10
Fraction Csp3	0.00
Num. rotatable bonds	0
Num. H-bond acceptors	3
Num. H-bond donors	1
Molar Refractivity	44.51
TPSA	50.44 Å²
**Lipophilicity**	
Log $P_{o/w}$ (iLOGP)	1.44
Log $P_{o/w}$ (XLOGP3)	1.58
Log $P_{o/w}$ (WLOGP)	1.50
Log $P_{o/w}$ (MLOGP)	1.04
Log $P_{o/w}$ (SILICOS-IT)	1.97
Consensus Log $P_{o/w}$	1.51

Water Solubility	
Log *S* (ESOL)	-2.46
Solubility	5.66e-01 mg/ml ; 3.49e-03 mol/l
Class	Soluble
Log *S* (Ali)	-2.25
Solubility	9.12e-01 mg/ml ; 5.62e-03 mol/l
Class	Soluble
Log *S* (SILICOS-IT)	-3.03
Solubility	1.53e-01 mg/ml ; 9.42e-04 mol/l
Class	Soluble
**Pharmacokinetics**	
GI absorption	High
BBB permeant	Yes
P-gp substrate	No
CYP1A2 inhibitor	Yes
CYP2C19 inhibitor	No
CYP2C9 inhibitor	No
CYP2D6 inhibitor	No
CYP3A4 inhibitor	No
Log K_p (skin permeation)	-6.17 cm/s
**Druglikeness**	
Lipinski	Yes; 0 violation
Ghose	No; 1 violation: #atoms<20
Veber	Yes
Egan	Yes
Muegge	No; 1 violation: MW<200
Bioavailability Score	0.55
**Medicinal Chemistry**	
PAINS	0 alert
Brenk	1 alert: cumarine
Leadlikeness	No; 1 violation: MW<250
Synthetic accessibility	2.56

Figure S14 Chemico-physical and pharmacokinetic properties predicted for umbelliferone.

eugenol

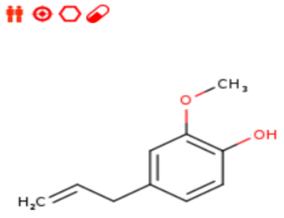

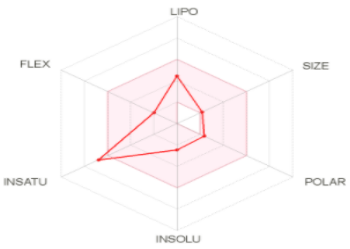

SMILES C=CCc1ccc(c(c1)OC)O

Physicochemical Properties	
Formula	C10H12O2
Molecular weight	164.20 g/mol
Num. heavy atoms	12
Num. arom. heavy atoms	6
Fraction Csp3	0.20
Num. rotatable bonds	3
Num. H-bond acceptors	2
Num. H-bond donors	1
Molar Refractivity	49.06
TPSA	29.46 Å²
**Lipophilicity**	
Log $P_{o/w}$ (iLOGP)	2.37
Log $P_{o/w}$ (XLOGP3)	2.27
Log $P_{o/w}$ (WLOGP)	2.13
Log $P_{o/w}$ (MLOGP)	2.01
Log $P_{o/w}$ (SILICOS-IT)	2.48
Consensus Log $P_{o/w}$	2.25

Water Solubility	
Log *S* (ESOL)	-2.46
Solubility	5.69e-01 mg/ml ; 3.47e-03 mol/l
Class	Soluble
Log *S* (Ali)	-2.53
Solubility	4.90e-01 mg/ml ; 2.98e-03 mol/l
Class	Soluble
Log *S* (SILICOS-IT)	-2.79
Solubility	2.65e-01 mg/ml ; 1.61e-03 mol/l
Class	Soluble
**Pharmacokinetics**	
GI absorption	High
BBB permeant	Yes
P-gp substrate	No
CYP1A2 inhibitor	Yes
CYP2C19 inhibitor	No
CYP2C9 inhibitor	No
CYP2D6 inhibitor	No
CYP3A4 inhibitor	No
Log K_p (skin permeation)	-5.69 cm/s
**Druglikeness**	
Lipinski	Yes; 0 violation
Ghose	Yes
Veber	Yes
Egan	Yes
Muegge	No; 1 violation: MW<200
Bioavailability Score	0.55
**Medicinal Chemistry**	
PAINS	0 alert
Brenk	1 alert: isolated_alkene
Leadlikeness	No; 1 violation: MW<250
Synthetic accessibility	1.58

Figure S15 Chemico-physical and pharmacokinetic properties predicted for eugenol.
